# Metabolomic analysis of serum from rats following long-term intake of Chinese sausage

**DOI:** 10.29219/fnr.v62.1447

**Published:** 2018-07-09

**Authors:** Minxian Rong, Pei Wang, Yuesheng Qiu, Yungang Liu, Yiyuan Wang, Hong Deng

**Affiliations:** 1 Department of Nutrition and Food Hygiene, Guangdong Provincial Key Laboratory of Tropical Disease Research, School of Public Health, Southern Medical University, Guangzhou, China; 2 Wuhan Centers for Disease Prevention and Control, Wuhan, China; 3 Department of Toxicology, Guangdong Provincial Key Laboratory of Tropical Disease Research, School of Public Health, Southern Medical University, Guangzhou, China

**Keywords:** Chinese sausage, metabolomics, NDMA, liver damage, GC-MS

## Abstract

**Introduction:**

Owing to the contamination of chemical pollutants, especially nitrosamines and their precursors, in Chinese sausage, long-term intake of Chinese sausage may have potential health effects.

**Objection:**

This study investigated the effects of long-term intake of Chinese sausage with different contaminations of *N*-nitrosodimethylamine (NDMA) on rat liver and the potential biomarkers in the serum.

**Methods:**

Serum metabolomic analysis was performed by gas chromatography–mass spectrometry at weeks 7, 17, 25, and 33; simultaneously, liver histopathological examination was conducted and its relationship with the serum metabolomics was also investigated.

**Results:**

In the study, long-term intake of Chinese sausage with different NDMA contents induced significant changes in serum metabolites and liver histopathology in rats. Metabonomic analysis showed that seven metabolites – β-alanine, 3-aminoisobutyric acid, aminooxyacetic acid, D-alanyl-D-alanine, pelargonic acid, palmitic acid (PA), and linoleic acid (LA) – in three sausage diet groups were significantly decreased at four time points, where three other metabolites were notably increased, which included putrescine, ethanolamine phosphate, and taurine. Among the various treatments, the NDMA (sausage-free) group demonstrated the most remarkable changes. Phenylalanine was decreased followed by an increase, and tyrosine persistently declined, both of which were elevated in the NDMA group. In addition, the histopathological result was consistent with that of the serum metabolomic analysis, and the changes in serum metabolites in each sausage diet group and the NDMA group were consistently associated with disorders of lipids, amino acid, and energy metabolism.

**Conclusion:**

This work indicates that excessive NDMA content in sausage may cause liver damage.

Chinese sausage is a famous traditional processed meat in China; the issue of its safety has been attracting more and more attention because of the contamination of chemical pollutants, especially nitrosamines and their precursors (nitrate, nitrite, etc.). In 2015, the International Agency for Research on Cancer (IARC) reported that processed meats had been classified as carcinogenic to humans (Group 1 carcinogen). The statement by the IARC confirmed that every intake of 50 g of processed meat per day might increase the risk of colorectal cancer by 18% (1). Many epidemiological studies indicate that consumption of smoked or processed meats and other nitrite-related foods is associated with increased risk of gastrointestinal, nasopharyngeal, and pancreatic tumors (2, 3). These consequences might be relevant to the presence of carcinogenic substances, such as volatile *N*-nitrosamines (VNAs) (4–6), polycyclic aromatic hydrocarbons (7), and heterocyclic amines produced during meat processing (pickling, smoking, or high temperature heating), in the diet (8).

Chinese sausage is different from Western dry fermented sausages in the manufacturing technology, seasoning, microbial ecology, and flavor, and the contamination of nitrosamines and their precursors in the former is supposed to be significant (9). Our previous studies found that the contamination of VNAs (including *N*-nitrosodimethylamine [NDMA], *N*-nitrosodiethylamine [NDEA], etc.) and its precursors in Chinese-style sausage was serious (9). NDMA formed in sausage might be mainly a result of reactions between dimethylamine and nitrite in certain conditions (10). Nitrite (nitrosating agents) is one of the vital precursors for the formation of NDMA. Its level in processed meat is influential on the concentration of NDMA. In addition, many other factors may also be influential on the formation of nitrosamines in sausages, such as the quality of raw meat used (e.g. microbial activity), use of additional additives (e.g. antioxidants and bioactivators), use of spices (e.g. anise and paprika), temperature during smoking processes, storage conditions (e.g. time and mode), and so on (11, 12). However, there is no way to completely prevent the formation of NDMA in sausages, and therefore NDMA in sausages has to be taken seriously in food safety.

In 1978, IRAC classified NDMA as a Group 2A carcinogen (probably carcinogenic to human) (13). The main target organ of toxicity of NDMA is the liver (14–16). NDMA has been administered to rodents in order to generate animal models of chronic hepatic damage and hepatocellular carcinoma (HCC) (14). It was also found that the histological and genetic signatures of NDMA-induced hepatocarcinogenesis are similar to those of human HCC. NDMA is converted into more toxic metabolites by cytochrome P450 enzymes in the liver (17), which simultaneously trigger the production of reactive oxygen species (ROS) as by-products (18, 19). Exposure of organisms to the toxic metabolites and elevated levels of ROS may result in liver damage. Hence, a subtle metabolic change in liver damage monitoring would be of great clinical importance.

The effects of foods on the body are very complex and subtle. It is difficult to accurately detect the bioactive compounds of foods and characterize their effects on the organism by conventional laboratory methods. Emerging platforms in systems biology provide a new methodology to identify subtle metabolic changes. Metabolomics is a particularly attractive technology that focuses on high-throughput identification and quantification of small-molecule (<1,500 Da) metabolites in a cell, organ, or organism (20). This approach has been used to identify serum principal metabolites and investigate the mechanism of exogenous material on organisms (21–24). In animal hepatotoxicity studies, toxic chemicals such as carbon tetrachloride (25), organophosphorus pesticide (26), and NDEA (27) significantly disrupted the level of several metabolites in blood, including arachidonic acid, lysophosphatidyl ethanolamines, and phosphatidylcholine. A metabolomic study of hepatotoxicity induced by chlorpyrifos and cadmium found that chlorpyrifos and cadmium could disrupt the energy, amino acid, and fatty acid metabolism in rat liver (28). A range of metabolites that represent liver dysfunctions have been examined in previous studies, suggesting the usefulness of metabolomics in exploring potential biomarkers of liver diseases caused by toxic chemicals.

Considering the contamination of nitrosamines and the precursors in Chinese sausages and their hepatotoxicity (such as that of NDMA), we speculate that long-term intake of Chinese sausages could cause hepatic damage, which might eventually progress to chronic liver disease, including hepatitis, cirrhosis, and so on. In the present study, the effects were investigated of long-term intake of different amounts of Chinese sausage and different NDMA contents in the sausage on rat liver, in particular at various time points as suggested by a model of NDMA-induced rat HCC. Moreover, a metabolic approach was applied to investigate the metabolic changes of metabolites in the serum of rats administered a sausage diet or NDMA by itself.

## Materials and methods

### Chemicals and reagents

All chemicals and solvents were of analytical or chromatographic grade. NDMA (99.0% purity) was obtained from Tokyo Century Chemical Co., Ltd. (Tokyo, Japan). Methanol, acetonitrile, pyridine, n-hexane, methoxylamine hydrochloride (97%), N,O-Bis(trimethylsilyl) trifluoroacetamide (BSTFA) with 1% Trimethylchlorosilane (TMCS) were purchased from CNW Technologies GmbH (Düsseldorf, Germany). L-2-chlorophenylalanine was from Shanghai Hengchuang Bio-technology Co., Ltd. (Shanghai, China).

### Animals and treatment

A total of 180 male Sprague Dawley rats weighing 180–220 g were obtained from the Laboratory Animal Center of Southern Medical University (Guangdong, China). All animal handlings were approved by the Animal Ethics Committee of Southern Medical University and were carried out in accordance with current Chinese legislation. All rats were allowed to acclimatize in communal iron cages for 2 weeks prior to treatment. They were kept at a controlled humidity (50–60%) and temperature (22 ± 2°C) with a 12 h light/dark cycle. After acclimatization, the rats were randomly assigned to five groups (*n* = 36/group): a control group (CON) fed with AIN-93G diet, positive control (NDMA) group fed with AIN-93G diet and 30 μg/kg NDMA in drinking water, and three sausage diet groups fed with sausage diets I, II, and III, respectively, for a treatment period as long as 33 weeks. Sausage diet I was a diet of 1 part Chinese sausage to 5.5 parts certified rat chow, and the concentration of NDMA in the sausage was 1.13 μg/kg, which was lower than the tolerance limit, specifically, 3 μg/kg, as established by the Ministry of Health of China. Sausage diet II was a diet of 1 part Chinese sausage to 5.5 parts certified rat chow, with a concentration of NDMA in the sausage of 7.37 μg/kg, which exceeded the tolerance limit. Sausage diet III was a diet of 1 part Chinese sausage to 2.7 parts certified rat chow, and the concentration of NDMA in the sausage was 7.37 μg/kg. For the NDMA group, NDMA was supplied to rats through drinking water containing 30 μg/kg NDMA for 12 weeks, at which point it was changed to sterile tap water. Other groups were given sterile tap water during the experiment. Sausage diets were modulated on a control diet of AIN-93G, including cornstarch, casein, maltodextrin, sucrose, soybean oil, fiber, mineral mix, vitamin mix, L-cysteine, choline bitartrate and tert-butylhydroquinone (Table 1). No significant energy or nutrient changes were found in any of the experimental group diets (*p* > 0.05). Moreover, all diets were commissioned by the Guangdong Medical Experimental Animal Center and were vacuum packed, cobalt 60 irradiated and sterilized, and stored at 4°C in the dark.

**Table 1:** Ingredients of rat diets per 100 g

Ingredients (g)	CON diet	Sausage diet I	Sausage diet II	Sausage diet III
Sausage*	–	18.0	18.0	36.0
Corn starch	39.7	39.7	39.7	39.7
Casein	20.0	15.6	16.2	12.4
Soybean oil	7.0	2.7	1.9	–
Sucrose	10.0	6.3	5.8	–
Maltodextrin	13.2	12.7	13.2	7.8
Mixed minerals	3.5	3.5	3.5	3.5
Mixed vitamins	1.0	1.0	1.0	1.0
L-cystine	0.3	0.3	0.3	0.3
Choline chloride	0.25	0.25	0.25	0.25
Fiber	5.0	5.0	5.0	5.0
Fat	7.0	7.3	7.3	10.8
Protein	18.2	19.1	19.0	18.2
Carbohydrates	60.3	63.3	62.1	57.8
Energy (kcal)**	377	389.3	390.1	401.2
Fat–energy ratio (%)	16.7	16.9	16.8	24.2
Protein–energy ratio (%)	19.3	19.6	19.5	18.1
Carbohydrate–energy ratio (%)	64.0	63.5	63.8	57.7

* Sausage diet I: the concentrations of NDMA in added sausage was 1.13 μg/kg, which did not exceed the tolerance limit of 3 μg/kg established by the Ministry of Health of China. Sausage diet II and sausage diet III: the concentrations of NDMA in added sausage was 7.37 μg/kg, which exceeded the tolerance limit of 3 μg/kg established by the Ministry of Health of China.

** 1 kcal = 4.184 kJ. NDMA, *N*-nitrosodimethylamine; CON, control.

According to our previous experiments involving a rat HCC model induced by drinking NDMA water, the serial progression of hepatocarcinogenesis in this animal model was divided into four stages: the inflammation stage (weeks 4–8), the fibrosis stage (weeks 9–17), the cirrhosis stage (weeks 18–25), and the HCC stage (weeks 26–33). The time points at week 7, week 17, week 25, and week 33 were the characteristic histological changes of the inflammation, fibrosis, cirrhosis, and HCC stages, respectively. Thus, eight rats from each group (*n* = 8) were randomly selected in the 7th, 17th, 25th, 33rd week after treatment and anesthetized with chloral hydrate via intraperitoneal injection. Blood samples were obtained from the aorta abdominalis before the rats were sacrificed. Serum was obtained through centrifugation at 3,000 rpm for 15 min; it was then transferred into Eppendorf tubes and stored at −80°C for gas chromatography–mass spectrometry (GC-MS) analysis. Specimens of liver were fixed in 10% neutral formaldehyde for histopathology analysis.

### Histopathology examination

The collected liver samples fixed in 10% neutral formaldehyde were paraffin-embedded, sectioned, and stained with hematoxylin–eosin. A microimaging system (Olympus; Tokyo, Japan) was used to observe the pathological changes. Microscopic examination of all liver tissues was done at the Pathology Laboratory of Southern Medical University. Histopathological scoring was performed in regard to the degree of hepatic inflammation and fibrosis, which was semi-quantified following the Ishak inflammation and fibrosis score system (29) (Tables S1 and S2 in the supplementary materials).

### Sample preparation for GC-MS analysis

Serum samples stored at −80°C were thawed at room temperature and then divided into aliquots of 50 μL. The sample was mixed with 10 μL of 2-chloro-l-phenylalanine dissolved in methanol as an internal standard and vortexed for 10 s. Subsequently, 150 μL of ice-cold mixture of methanol–acetonitrile (2/1, v/v) was added, and the mixture was vortexed for 1 min, ultrasonicated at ambient temperature (25–28°C) for 5 min, and stored at −20°C for 10 min. The extract was centrifuged at 12,000 rpm, 4°C for 10 min, and the supernatant (150 μL) was collected in a glass vial and dried in a freeze concentration centrifugal dryer. Eighty microliters of 15 mg/ml methoxylamine hydrochloride in pyridine was subsequently added. The resultant mixture was vortexed vigorously for 2 min and incubated at 37°C for 90 min. Finally, 80 μL of BSTFA (with 1% TMCS) and 20 μL n-hexane were added into the mixture, and the samples were vortexed vigorously for 2 min and then derivatized at 70°C for 60 min and placed at ambient temperature for 30 min before GC-MS analysis. To ensure the stability and repeatability of the GC-MS systems, pooled quality control (QC) samples were used. The QC samples were prepared from a mixture of all sample extracts, treated and tested in the same way as the sample analyzed. One QC sample was inserted and analyzed for every 10 samples.

### GC-MS nontargeted metabolism analysis

GC-MS analysis was carried out on a gas chromatograph system (Agilent J&W Scientific, Folsom, CA, California, USA, model 7890B) coupled with a mass selective detector (Agilent, model 5977A). A DB-5MS fused-silica capillary column (30 m × 0.25 mm × 0.25 μm; Agilent) was utilized to separate the derivatives. The analysis was performed under the following conditions: helium (>99.999%) was used as the carrier gas at a constant flow rate of 1 ml/min through the column. The injector temperature was maintained at 260°C. Injection volume was 1 μL by splitless mode, and the solvent delay time was set to 5 min. The initial oven temperature was 50°C; it was ramped to 125°C at a rate of 15°C/min, to 210°C at a rate of 5°C/min, to 270°C at a rate of 10°C/min, and to 305°C at a rate of 20°C/min and finally held at 305°C for 5 min. The temperature of the MS quadrupole and electron impact ion source was set to 150°C and 230°C, respectively. Ions were generated by 70 eV electron energy at a full scan mode (m/z 50–60).

### Data analysis

Statistical analysis was performed by one-way analysis of variance using SPSS version 20.0 (Beijing Stats Data Mining Co., Ltd., China); *p* < 0.05 was considered statistically significant.

The GC-MS raw data were analyzed by Chroma Time-of-Flight (TOF) software (v. 4.34, LECO, St Joseph, MI, USA) and LECO-Fiehn Rtx5 database for data pretreatment procedures, such as data baseline filtering and calibration, peak alignment, deconvolution analysis, peak identification, and peak area integration. Then metabolites from the GC-MS spectra were identified with a similarity more than 400. The resulting data were imported into SIMCA-P 13.0 (Umetrics, Umea, Sweden) where a nonsupervised principal component analysis (PCA) was used to visualize general clustering change trends or outliers among the observations. To further distinguish the overall differences in metabolic profiles among the groups, orthogonal partial least squares–discriminant analysis (OPLS-DA) models were utilized. The parameters of OPLS-DA (R^2^Y, Q^2^Y) were used for the evaluation of the models. The goodness of fit and predictive ability of the models was quantified as the values of R^2^Y and Q^2^Y (30). Sevenfold cross validation and a response permutation test were applied to guard against OPLS-DA model overfitting (Supplementary materials, Fig. S2).

Potential biomarkers were selected based on the variable importance in the projection (VIP). Those metabolites with a VIP value greater than 1 in the first principal component of the OPLS-DA model were analyzed, among which each comparison with the control yielding a *p*-value by the Student’s *t*-test lower than 0.05 defined a differential metabolite. Then the demonstration of these metabolites, as observed in this study, were matched with their structure messages, which were obtained from the Human Metabolome Databases (HMDB). Finally, the key metabolites were investigated for their metabolism pathways using the databases of Kyoto Encyclopedia of Genes and Genomes (KEGG) (http://www.kegg.jp/) and HMDB (http://www.hmdb.ca).

## Results and discussion

### Body weights

**Fig. 1:**
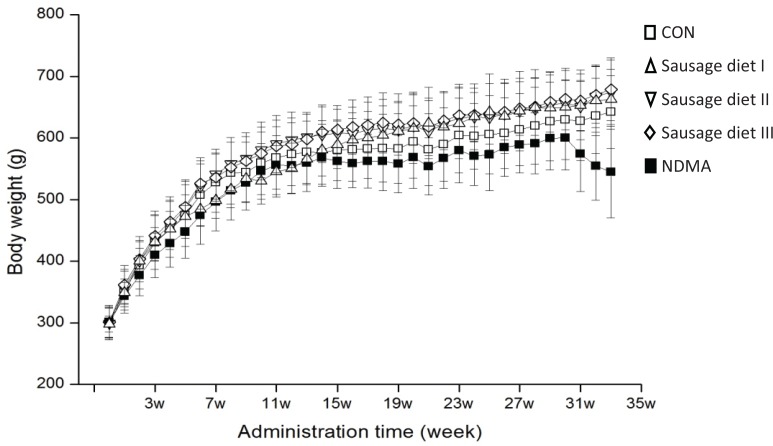
Body weights of rats were measured during the experiment. It’s expressed as mean ± S.D. Open square: fed with AIN-93G diet (CON group); regular triangle: fed a granular diet of 1 part Chinese sausage (NDMA content in sausage not exceeding the tolerated limit) to 5.5 parts certified rat chow (sausage diet I); inverted triangle: fed a granular diet of 1 part Chinese sausage (excessive NDMA content in sausage) to 5.5 certified rat chow (sausage diet II); rhombus: fed a granular diet of 1 part Chinese sausage (excessive NDMA content in sausage) to 2.7 parts certified rat chow (sausage diet III); filled square: fed with AIN-93G diet and 30 μg/kg NDMA in drinking water (NDMA group). NDMA, *N*-nitrosodimethylamine; CON, control.

The body weights (BWs) of all rats after long-term intake of Chinese sausage were recorded (Fig. 1). The BWs of rats in three sausage diet groups at different time points did not show any significant changes compared with the CON group, as did feeding and water consumption (data not listed). However, changes occurred in BW between NDMA group and the other four groups after 16 weeks (*p* < 0.05): a dramatic decrease was observed in the NDMA group from the 31st week to the end. This showed that drinking water containing 30 μg/kg NDMA impaired the health of rats.

### Liver histopathology

**Fig. 2:**
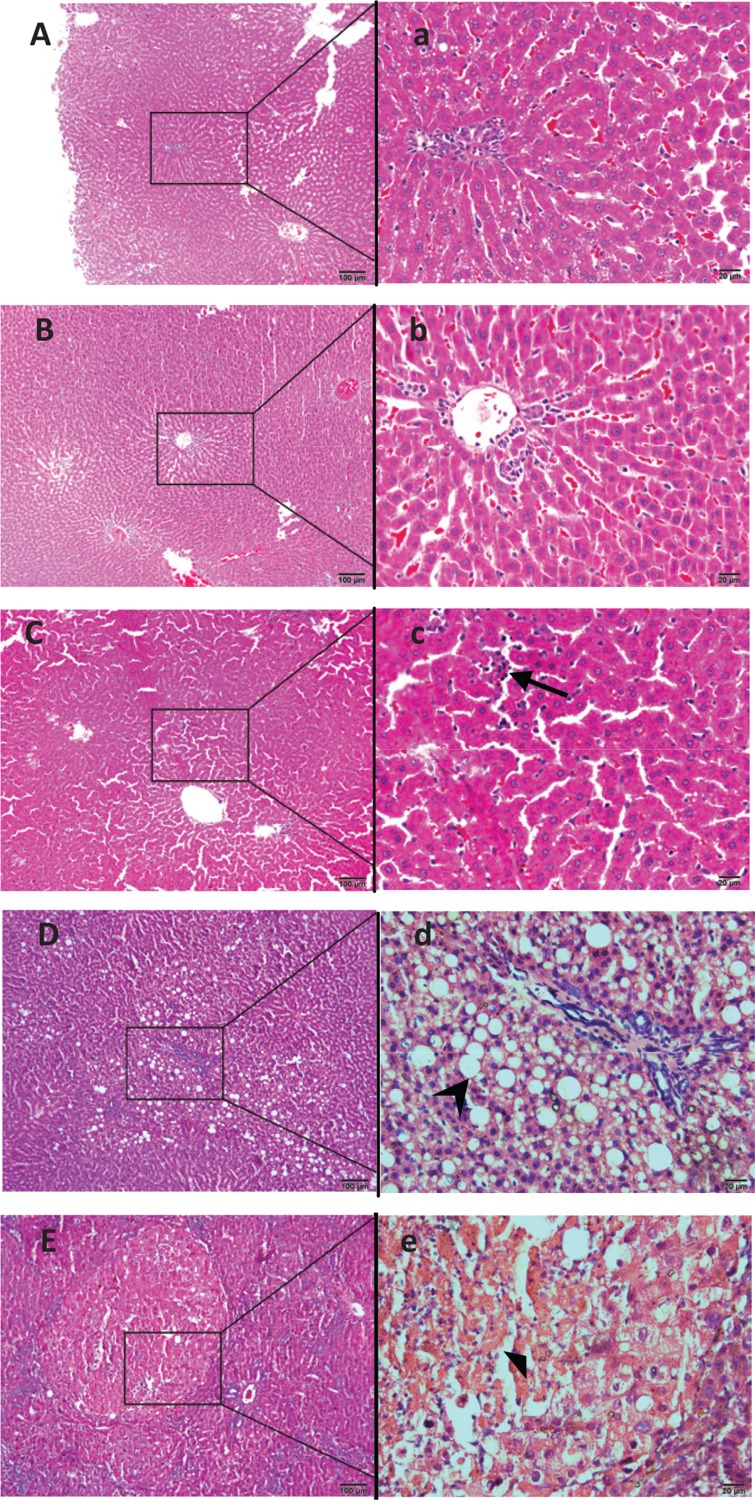
Representative photomicrographs of H&E-stained formalin fixed liver sections from each group rats. During the 33 weeks of feeding, normal liver histology was shown in the control group (A, ×100; a, ×400). A slight hepatic sinusoid was found in sausage diet group I after 25 weeks of feeding (B, ×100; b, 400). In the meantime, a moderate hepatic sinusoidal dilatation, infiltration of a large amount of inflammatory cells (arrow) in hepatic lobules were observed in sausage diet group II (C, ×100; c, ×400). Moreover, fatty degeneration (arrow), fibroblastic proliferation in the portal area, and hepatic lobules were present in sausage diet group III (D, ×100; d, ×400). For the NDMA group, the hepatocyte necrosis (solid triangle), as indicated by the swollen and pale-staining hepatocytes with dilated endoplasmic reticulum, occurred after 25 weeks (E, ×100; e, ×400). NDMA, *N*-nitrosodimethylamine; H&E, hematoxylin–eosin.

Through pathological examination, with semi-quantitative scoring, of the liver tissues from rats in each group, it was evidenced that long-term intake of different amounts of Chinese sausage or intake of sausage with different NDMA contents resulted in varying degrees of liver damage in rats. In the CON group, the hepatic lobules of the rats were complete and clear, the hepatic cords were neat, and hepatocytes were normal during the whole experiment (Fig. 2A). The pathological changes in the liver, including inflammation and fibrosis, both scored as described in Section 2.3, in the three sausage diet groups were specific for the particular feeding time points, the amount of sausage in the feed, and the concentration of NDMA in the sausage, as shown in Fig. 2B through D and Table 2. The liver inflammation score increased along with the elevation of NDMA content in the sausage diet, that is, from sausage diet groups I to III. The score of fibrosis in sausage diet group III was higher than that in the CON group at week 33 (*p* < 0.05), while it was near 0 in sausage diet groups I and II, without statistical significance when compared with the CON group. In the NDMA (sausage-free) group, the pathological changes in the liver were more severe than those in the sausage groups containing NDMA, at lower levels, with the highest scores of liver inflammation and fibrosis as shown in Fig. 2E and Table 2. Clearly, the results of this study suggest that long-term intake of Chinese sausage with excessive NDMA content may cause liver damage.

**Table 2:** Scores of liver inflammation and fibrosis in each group according to the Ishak score system

Groups	Liver inflammation score					Fibrosis score				
	7 weeks	17 weeks	25 weeks	33 weeks	*p*	7 weeks	17 weeks	25 weeks	33 weeks	*p*
CON	0 ± 0	0 ± 0	0.5 ± 0.7	1.3 ± 0.9	0.000	0 ± 0	0 ± 0	0 ± 0	0 ± 0	–
Sausage diet I	0.3 ± 0.7	0.3 ± 0.7	0.7 ± 0.9	2.4 ± 0.9*	0.000	0 ± 0	0 ± 0	0 ± 0	0 ± 0	–
Sausage diet II	3.9 ± 0.8*^a^	7.4 ± 0.8*^a^	9.4 ± 0.8*^a^	12.6 ± 0.8*^a^	0.000	0 ± 0	0 ± 0	0 ± 0	0.3 ± 0.9	–
Sausage diet III	8.0 ± 0.9*^b^	10.9±1.0*^b^	12.5 ± 1.0*^b^	15.9 ± 0.9*^b^	0.000	0 ± 0	0.2 ± 0.7	0.7 ± 0.9	2.1 ± 0.6*	0.000
NDMA	11.1 ± 1.0*	14.3±0.9*	15.4 ± 0.9*	16.9 ± 1.0*	0.164	0 ± 0	2.8 ± 0.6*	3.9 ± 0.7*	4.7 ± 0.8*	0.001

Values are means ± S.D. Statistical significance was observed when compared with

* the CON group,

a sausage diet group I, and

b sausage diet group II. NDMA, N-nitrosodimethylamine; CON, control.

### GC-MS analysis

#### GC-MS fingerprinting and multivariate analysis

All samples were analyzed by GC-MS in full scan mode to obtain serum metabolic profiles containing as many compounds as possible. The typical GC-MS total ion chromatograms (TICs) of rat serum were shown in Fig. S2 in the supplementary materials. In this study, the great stability and reproducibility of the retention time and the intensity of the chromatographic peaks in the QC and all groups indicated that the whole analysis method, including pretreatment and the GC-MS system, was stable and reliable.

**Fig. 3:**
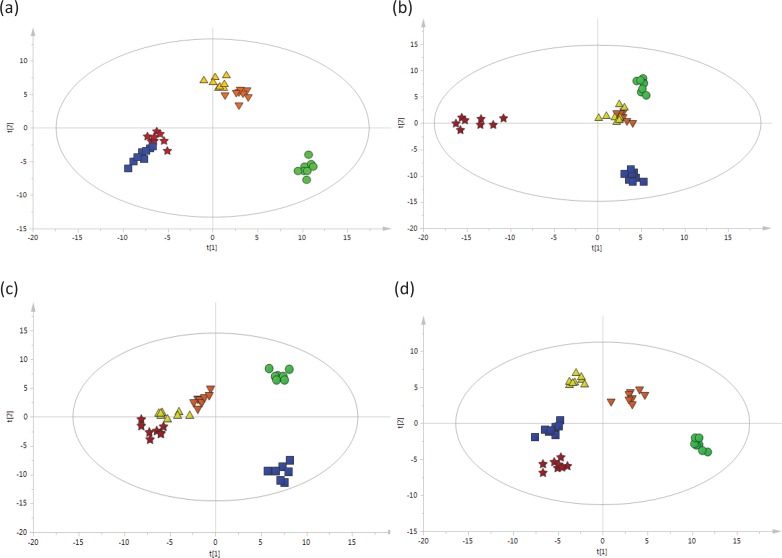
OPLS-DA score plots (a through d) based on the GC-MS data of rat serum treated with sausage and NDMA at different treatment times: (a) rats treated for 7 weeks; (b) rats treated for 17 weeks; (c) rats treated for 25 weeks; (d) rats treated for 33 weeks; *n* = 8 per group. Green filled circles: given the AIN-93G diet (CON group); blue filled squares: fed a granular diet of 1 part Chinese sausage (NDMA content in sausage not exceeding the tolerated limit) to 5.5 parts certified rat chow (sausage diet I); orange filled inverted triangles: fed a granular diet of 1 part Chinese sausage (excessive NDMA content in sausage) to 5.5 parts certified rat chow (sausage diet II ); yellow filled triangles: fed a granular diet of 1 part Chinese sausage (excessive NDMA content in sausage) to 2.7 parts certified rat chow (sausage diet III); red filled pentacles: fed with the AIN-93 diet and 30 μg/kg NDMA in the drinking water group (NDMA group). NDMA, *N*-nitrosodimethylamine; OPLS-DA, orthogonal partial least squares–discriminant analysis; GC-MS, gas chromatography–mass spectrometry.

After the peak detection and alignment of all TICs, a total of 253 metabolites were enrolled in the final data set for the statistical analysis. To model and evaluate the systemic changes in the metabolites in rat serum, the obtained data were analyzed by PCA and OPLS-DA for each time point. The resultant plots are shown in Fig. S3 in the supplementary materials and Fig. 3a through d. For OPLS-DA plots, the plots for the three sausage diet groups and the NDMA group clearly deviated from that of the CON group in the 7th week (Fig. 3a). And the plots for the three sausage diet groups separated from the NDMA group in the 25th week (Fig. 3c) and completely deviated in the 33rd week of treatment (Fig. 3d). These plots suggested that treatment with long-term intake of Chinese sausage or NDMA induced prominent changes in serum metabolites. The plots for the three sausage diet groups did not completely deviate at week 17 (Fig. 3b), while a clear separation was found at week 25 (Fig. 3c), which suggested that treatment with different amount of sausage in the feed and different concentrations of NDMA in the sausage could induce different changes in serum metabolites. The performance parameters for the classification calculated from the software were R^2^Y = 0.956, Q^2^ = 0.732 for 7 weeks of treatment (Fig. 3a); R^2^Y = 0.958, Q^2^ = 0.786 for 17 weeks of treatment (Fig. 3b); R^2^Y = 0.928, Q^2^ = 0.706 for 25 weeks of treatment (Fig. 3c); R^2^Y = 0.971, Q^2^ = 0.73 for the 33 weeks (Fig. 3d), which indicated that these models had good fitness and predictions. As results of the permutation test, the R^2^Y-intercept was 0.735, 0.713, 0.621, 0.789; and the Q^2^-intercept was −0.436, −0.452, −0.44, −0.468 (Supplementary materials, Fig. S1). Apparently, no overfitting of the data was present in our study.

#### Differential metabolites

Serum metabolites in rats changed significantly during the early feeding stage (week 7) in the three sausage groups, including sausage diet I, which contained low amounts of sausage with low content of NDMA in the feed. However, no pathological changes in rat liver were observed in the early stage (week 7) in the three sausage groups, except the NDMA group. Thus, the changes of serum metabolites might be more sensitive than the liver histopathological examination in detecting physiological response to toxicants. The metabolic profiles of the treated groups and the CON group deviated clearly via OPLS-DA (Fig. 3), changes that followed exposure to different amount of sausage and different concentrations of NDMA at different time points. Thirteen metabolites that notably contributed to this discrimination were identified (Table 3). β-Alanine, 3-aminoisobutyric acid, aminooxyacetic acid, D-alanyl-D-alanine, pelargonic acid, PA, and LA were decreased in the three sausage diet groups and the NDMA group, while putrescine, ethanolamine phosphate, and taurine were increased. Phenylalanine was firstly decreased at week 7 following by an increase in the 33rd week in the three sausage diet groups. Phenylalanine, tyrosine, and α-ketoglutarate were elevated over all time periods in the NDMA group. With metabolomics analysis of serum, some of these metabolites were found to play an important role in the metabolic pathways of lipids, amino acids, and energy. The pathogenesis of liver diseases may be attributable to metabolic changes, reflected by abnormal body lipids, amino acids, and energy metabolism in the serum (31).

**Table 3:** Change in the serum metabolites from rats treated with sausage and NDMA compared with control group for different time points and doses

Number	Metabolites	Molecular formula	Mass^a^	RT^b^	Groups	Fold change^c^			
						7th week	17th week	25th week	33rd week
1	β-Alanine	C_3_H_7_NO_2_	102	5.17	Sausage diet I	0.50**	0.36**	0.50**	0.52**
					Sausage diet II	0.77*	0.67**	0.61*	–
					Sausage diet III	0.59*	0.53**	0.50**	0.65**
					NDMA	0.49**	0.45**	0.56**	–
2	3-aminoisobutyric acid	C_4_H_9_NO_2_	102	5.68	Sausage diet I	0.73**	0.67**	0.59**	–
					Sausage diet II	0.66**	0.63**	0.50*	0.75**
					Sausage diet III	0.43**	0.49**	0.39**	0.57**
					NDMA	0.23**	0.25**	–	0.24
3	Aminooxyacetic acid	_	160	6.12	Sausage diet I	–	0.67**	–	–
					Sausage diet II	–	–	0.71*	–
					Sausage diet III	–	–	0.64*	0.76*
					NDMA	0.75*	0.73*	0.65*	0.48**
4	D-alanyl-D-alanine	C_6_H_12_N_2_O_3_	96	7.51	Sausage diet I	–	0.77*	–	0.54*
					Sausage diet II	–	–	–	–
					Sausage diet III	–	–	0.72*	0.83*
					NDMA	–	0.81*	0.62*	0.63*
5	Pelargonic acid	C_9_H_18_O_2_	118	9.38	Sausage diet I	–	0.68*	–	–
					Sausage diet II	0.68*	–	–	0.79*
					Sausage diet III	–	0.72*	–	0.68*
					NDMA	0.69*	0.49*	0.71*	0.66*
6	Putrescine	C_4_H_12_N_2_	86	11.54	Sausage diet I	3.95**	2.81**	4.44**	3.73**
					Sausage diet II	2.19**	1.68**	0.00**	1.29*
					Sausage diet III	2.79**	2.26**	1.48**	1.60*
					NDMA	3.76**	2.93**	1.92**	2.04**
7	α-Ketoglutarate	C_5_H_6_O_5_	203	13.51	Sausage diet I	–	–	–	1.42*
					Sausage diet II	–	0.70**	–	–
					Sausage diet III	–	–	1.30*	0.58**
					NDMA	–	1.03*	1.83*	2.37**
8	Phenylalanine	C_9_H_11_NO_2_	192	14.54	Sausage diet I	–	–	0.84*	–
					Sausage diet II	–	0.86**	0.81*	1.13*
					Sausage diet III	–	–	0.85*	1.12*
					NDMA	–	1.23**	1.35**	1.75**
9	Ethanolamine phosphate	C_2_H_8_NO_4_P	299	15.22	Sausage diet I	2.47*	3.17*	3.08*	3.66*
					Sausage diet II	1.97*	1.37*	–	2.15**
					Sausage diet III	–	2.28**	2.11**	2.53*
					NDMA	3.63**	2.95**	2.86**	3.97**
10	Taurine	C_2_H_7_NO_3_S	188	15.34	Sausage diet I	2.07**	2.66**	1.58*	1.51*
					Sausage diet II	1.99**	1.60**	1.55*	1.38*
					Sausage diet III	2.86**	1.73*	2.11**	1.55*
					NDMA	2.81**	2.35**	2.94**	2.21**
11	Tyrosine	C_9_H_11_NO_3_	218	20.86	Sausage diet I	–	0.84*	0.75*	0.83*
					Sausage diet II	0.83*	0.79*	0.81*	0.76**
					Sausage diet III	0.86*	0.79*	0.77*	0.81*
					NDMA	–	–	1.25*	2.14**
12	Palmitic acid	C_16_H_32_O_2_	118	22.89	Sausage diet I	–	0.64**	0.66*	0.62**
					Sausage diet II	–	0.76*	0.76*	0.72*
					Sausage diet III	0.63**	0.71*	0.60**	0.72*
					NDMA	0.58**	0.71*	0.67*	0.73*
13	Linoleic acid	C_4_H_4_O_5_	82	25.19	Sausage diet I	–	0.52**	0.31**	0.32**
					Sausage diet II	–	0.59**	0.47**	0.38**
					Sausage diet III	0.45**	0.42**	0.31**	0.35**
					NDMA	0.69*	–	–	–

a Calculation mass;

b RT: retention time (minutes);

* significantly increased or decreased when compared with the control group at *p* < 0.05;

** significantly increased or decreased when compared with the control group at *p* < 0.001;

c relative concentration of treated rats to the control at the same time. NDMA, N-nitrosodimethylamine; CON, control.

Four differential metabolites, including PA, LA, taurine, and ethanolamine phosphate, were identified for the treated groups. These four substances in serum are closely related to the body lipid metabolism, suggesting that exposure to Chinese sausage or NDMA could affect the lipid metabolism of rats. PA and LA declined in the 7th week in sausage diet group III and the NDMA group, while they decreased in the 17th week both in sausage diet group I and sausage diet group II. Both of them are free fatty acids (FFA). It has been reported that changes of FFA have damaging effects, such as affecting cell proliferation, significantly increasing hepatotoxicity characterized by liver degeneration, inflammatory cell infiltration, and promoting the formation of liver fibrosis (32–34). The decrease of PA and LA may cause lipid metabolism turbulence in the liver and result in lipogenesis in rat hepatocytes. LA is also an essential fatty acid with important physiological functions such as regulating blood lipids and participating in the synthesis of phospholipids. Its reduction may lead to skin damage, liver and kidney diseases, and so on. It has been found that the reduction of LA results in steatosis and lipid metabolism disorders in rat hepatocytes and leads to liver damage (33, 35). In addition, if LA is insufficient, cholesterol will combine with certain saturated fatty acids, causing metabolic disturbances, depositing in the blood vessel wall, gradually leading to atherosclerosis, and triggering cardiovascular and cerebrovascular diseases.

Taurine plays a vital role in the metabolism of bile acid. It can combine anthropodesoxycholic acid under the action of amino acid N-acyltransferase to form taurochenodeoxycholic acid, which is excreted as a component of the bile into the alimentary canal. It functions to promote the digestion and absorption of lipids and fat-soluble vitamins (36). Liver damage, especially chronic liver diseases, can be reflected by an increase of blood bile acids (37). In this study, a significant increase of taurine in rat serum was found following administration of sausage diet or NDMA drinking water, which was consistent with the study of non-alcoholic fatty liver disease (38). Ethanolamine phosphate, a major source of the end-product of sphingosine degradation, was elevated significantly at week 7 after the administration of sausage diets or NDMA drinking water, but the latter changed more dramatically. According to the sphingosine biology pathway, this result might exacerbate hepatic metabolic disorders in the sausage diet groups and NDMA group (39, 40). The liver plays an important role in lipid metabolism, including both the endogenous and exogenous circulation of lipid metabolism and the lipid transport through the serum. Loss of liver function as caused by some hepatotoxicity may disrupt lipid metabolism (41, 42).

In the current study, α-ketoglutarate, phenylalanine, tyrosine, and putrescine were significantly changed in the sausage diet groups and NDMA group compared with the control. Numerous studies have reported the dysregulation of amino acid metabolism in liver disease (43, 44). As an essential catalytic reaction enzyme of amino acid, asparagine aminotransferase (AST) mainly affects the levels of aspartate, α-ketoglutarate, tyrosine, phenylalanine, oxaloacetate, and glutamate. AST preferentially promotes metabolism of aspartate, phenylalanine, tyrosine, cysteine, and so on, serving as −NH_2_ donors in the transamination of α-ketoglutarate to glutamate (45). It is commonly known that serum AST is a biomarker of liver damage (46). As shown in Fig. S4 (in the supplementary materials), the levels of serum AST in sausage diet group III and the NDMA (sausage-free) group were increased significantly (as compared with the CON group); this is consistent with the changes in serum α-ketoglutarate, phenylalanine, and tyrosine metabolism, as observed in our metabolomics study.

α-Ketoglutarate, a crucial metabolic intermediate of energy production, can be converted to succinyl-coenzyme A (succinyl-CoA) and shunted to the tricarboxylic acid (TCA) cycle. Succinyl-CoA is phosphorylated by succinyl-CoA synthetase accompanied with the hydrolysis of the energy-rich thioester bond for cell growth and proliferation (31). In the present work, α-ketoglutarate was remarkably increased in the NDMA group, suggesting that NDMA-induced liver damage might indirectly lead to imbalance of the TCA cycle; this might be caused by dysregulation of amino acid metabolism in hepatic dysfunction. Another observation related to AST catalysis in the present study was the concentration of phenylalanine and tyrosine, which was changed in both the sausage groups and the NDMA group during the whole experiment. Phenylalanine and tyrosine are aromatic amino acids and their metabolisms are influenced by AST. In the sausage groups phenylalanine went down, followed by a recovery; tyrosine declined persistently, while both amino acids were elevated in the NDMA group. The mechanism and significance of these changes in our study needs further investigation.

Putrescine was significantly increased in the three sausage diet groups as well as in the NDMA group. Putrescine is a polyamine; excessive amounts in vivo are closely related to cell growth and cancer and are likely to be involved in the regulation of genetic processes from DNA synthesis to cell migration, proliferation, differentiation, and apoptosis (47). According to reports in the past, an elevation of putrescine in this study might be related to dysfunction of the liver resulting from NDMA toxicity or polyamines preserved in sausage. However, more insightful research is required to explore how NDMA induces hepatotoxicity by the level of putrescine in vivo.

## Conclusions

In this study, liver damage in rats was observed following long-term exposure to Chinese sausage diets, especially that containing a high level of NDMA. In both the sausage diet groups and the NDMA group the metabolite changes in serum were predominantly disorders of lipids, amino acids, and energy metabolism. The histopathological changes and metabolic signatures related to high level NDMA in sausage were particularly similar to those in the NDMA group. Therefore, excessive NDMA levels in sausage may contribute to liver damage.
